# Utilizing a user-centered approach to develop and assess pharmacogenomic clinical decision support for thiopurine methyltransferase

**DOI:** 10.1186/s12911-019-0919-4

**Published:** 2019-10-17

**Authors:** Khoa A. Nguyen, Himalaya Patel, David A. Haggstrom, Alan J. Zillich, Thomas F. Imperiale, Alissa L. Russ

**Affiliations:** 1 Department of Pharmacotherapy and Translational Research, University of Florida, College of Pharmacy, 1225 Center Drive, Gainesville, FL 32610 USA; 2 Center for Health Services Research, Regenstrief Institute Inc., 1101 W 10th St, Indianapolis, IN USA; 3 Center for Health Information and Communication, Department of Veterans Affairs (VA), Veterans Health Administration, Health Services Research and Development Service (CIN 13-416), Richard L. Roudebush VA Medical Center, 1481 W 10th St, Indianapolis, IN 46202 USA; 4 Department of Pharmacy Practice, College of Pharmacy, Purdue University, 640 Eskenazi Avenue, Indianapolis, IN USA; 5 Department of Medicine, Indiana University School of Medicine, Indianapolis, IN USA

**Keywords:** Pharmacogenomics, Clinical decision support, User-centered approach, TPMT, Formative evaluation

## Abstract

**Background:**

A pharmacogenomic clinical decision support tool (PGx-CDS) for thiopurine medications can help physicians incorporate pharmacogenomic results into prescribing decisions by providing up-to-date, real-time decision support. However, the PGx-CDS user interface may introduce errors and promote alert fatigue. The objective of this study was to develop and evaluate a prototype of a PGx-CDS user interface for thiopurine medications with user-centered design methods.

**Methods:**

This study had two phases: In phase I, we conducted qualitative interviews to assess providers’ information needs. Interview transcripts were analyzed through a combination of inductive and deductive qualitative analysis to develop design requirements for a PGx-CDS user interface. Using these requirements, we developed a user interface prototype and evaluated its usability (phase II).

**Results:**

In total, 14 providers participated: 10 were interviewed in phase I, and seven providers completed usability testing in phase II (3 providers participated in both phases). Most (90%) participants were interested in PGx-CDS systems to help improve medication efficacy and patient safety. Interviews yielded 11 themes sorted into two main categories: 1) health care providers’ views on PGx-CDS and 2) important design features for PGx-CDS. We organized these findings into guidance for PGx-CDS content and display. Usability testing of the PGx-CDS prototype showed high provider satisfaction.

**Conclusion:**

This is one of the first studies to utilize a user-centered design approach to develop and assess a PGx-CDS interface prototype for Thiopurine Methyltransferase (TPMT). This study provides guidance for the development of a PGx-CDS, and particularly for biomarkers such as TPMT.

## Background

Pharmacogenomics, the study of the effect of genetic variation on drug response, is being studied more extensively and is receiving more attention from both clinicians and researchers [1, 2]. Providers’ incorporation of pharmacogenomic results into prescribing decisions can improve both treatment efficacy and safety by accounting for the predicted drug response of individual patients [2–4]. The pharmacogenomic biomarker for thiopurine methyltransferase (TPMT) has been studied extensively due to the drug’s serious side effects, such as neutropenia and myelosuppression [5–8]. Patients with leukemia or inflammatory bowel disease (IBD) who carry a TPMT mutation, especially those with biallelic mutations, can experience myelosuppression when undergoing treatment with thiopurine medications such as azathioprine and mercaptopurine. Myelosuppression is a potentially dangerous decrease in the production of white blood cells that can lead to death [9]. Therefore, testing for a TPMT mutation has been recommended before starting thiopurine therapy [10–12].

An essential factor for successful pharmacogenomic implementation into clinical practice is the delivery of information via clinical decision support (CDS) tools. CDS can be incorporated into the electronic health record (EHR) through an interruptive alert, or via other mechanisms as part of a patient record. Pharmacogenomic clinical decision support (PGx-CDS) tools for TPMT-based therapy may improve implementation of pharmacogenomics by providing up-to-date, evidence-based guidelines and real-time decision support.

Many health care systems are starting to implement PGx-CDS for several biomarkers [10, 13–15]. Within the last decade, 16 different PGx-CDSs have been developed and implemented into EHRs to guide pharmacogenomic practice [16]. However, many PGx-CDSs appear to have been developed without a formal assessment of clinicians’ needs or a systematic set of design requirements. Clinical data, including genetic results, lab values, diagnoses, patient information, and drug interactions, are all variables used to make prescribing decisions. Since these data are complex and are delivered in a fast-paced environment, CDS guidance needs to be organized and presented effectively to aggregate diffuse information for prescribers and aid their medication prescribing decisions [15, 17]. In addition, poor interface design of PGx-CDS can introduce errors and promote alert fatigue, hindering the effectiveness of pharmacogenomic prescribing [15, 18]. Therefore, the objective of this study was to apply user-centered design methods to develop and evaluate a prototype of a PGx-CDS user interface for thiopurine medications to assist providers with their prescribing decisions.

## Methods

### Study design and setting

This study was conducted at a large, midwestern Veterans Affairs (VA) Medical Center and a co-located, major academic healthcare system (MAHS). For EHRs, the VA uses the Computerized Patient Record System (CPRS) while the MAHS uses a commercial EHR. At the time of this study, neither healthcare system used any PGx-CDS in their prescribing processes.

This study was conducted in two phases. In Phase I, we conducted qualitative interviews to assess the information needs of physicians to develop interface design requirements for PGx-CDS for TPMT. Phase I interviews were conducted at each physician’s facility. Results from Phase I were applied in Phase II to develop a prototype and evaluate the design of PGx-CDS through usability testing with physicians. Phase II usability testing was performed at the VA Health Services Research and Development (HSR&D) Human-Computer Interaction and Simulation Laboratory [19] or via remote usability testing, based on participants’ preferences. This study was approved by the Indiana University IRB (protocol 1602878929R003.)

### Phase I: qualitative interview

#### Participant recruitment

Physicians from both healthcare systems were recruited for a 30-min, semi-structured interview conducted by a pharmacist researcher (KN). Physicians were eligible to participate if they had prescribed azathioprine or mercaptopurine in their clinical practice. Participating physicians were divided into two groups of specialty pracitce: gastroenterology and oncology with each group further divided into subspecialty of adult and pediatric practice. Each participant was verbally consented prior to the interview (the IRB approved the exemption for written consent for this phase).

#### Procedure and data collection

Participants were interviewed individually. The full interview guide is available in the Additional file 1: S4. Interview questions. Examples of questions include:

1. *What is your perception and interpretation of the meaning of TPMT pharmacogenomic clinical decision support tools?*
2. *If the computer system provided decision support for pharmacogenomics, would you use it in your clinical practice? Why or why not?*
  - *How might such a tool be helpful to you?*
  - *What barriers do you foresee for incorporating the tool into your work?*
  - *Would you like such a system to work similarly to an alert system?*
  - *What information would you like to see presented in a PGx-CDS?*

Interviews were audio recorded and transcribed for analysis [20]. Two independent analysts, a pharmacist researcher, and a human-computer interaction expert analyzed transcripts through a combination of inductive and deductive qualitative analysis to identify initial theme [21]. A priori codes were derived from the interview questions. Analysts met regularly to discuss and agree upon themes that were identified inductively. Similar to other literature, analysts independently coded the transcripts then met weekly to discuss discrepancies until reaching consensus [22]. A third individual, a human factors expert, was consulted if discrepancies could not be resolved. Data were analyzed and managed using NVivo 10 (QSR International).

### Phase II: prototype development and usability testing

#### Prototype development

**Fig. 1:**
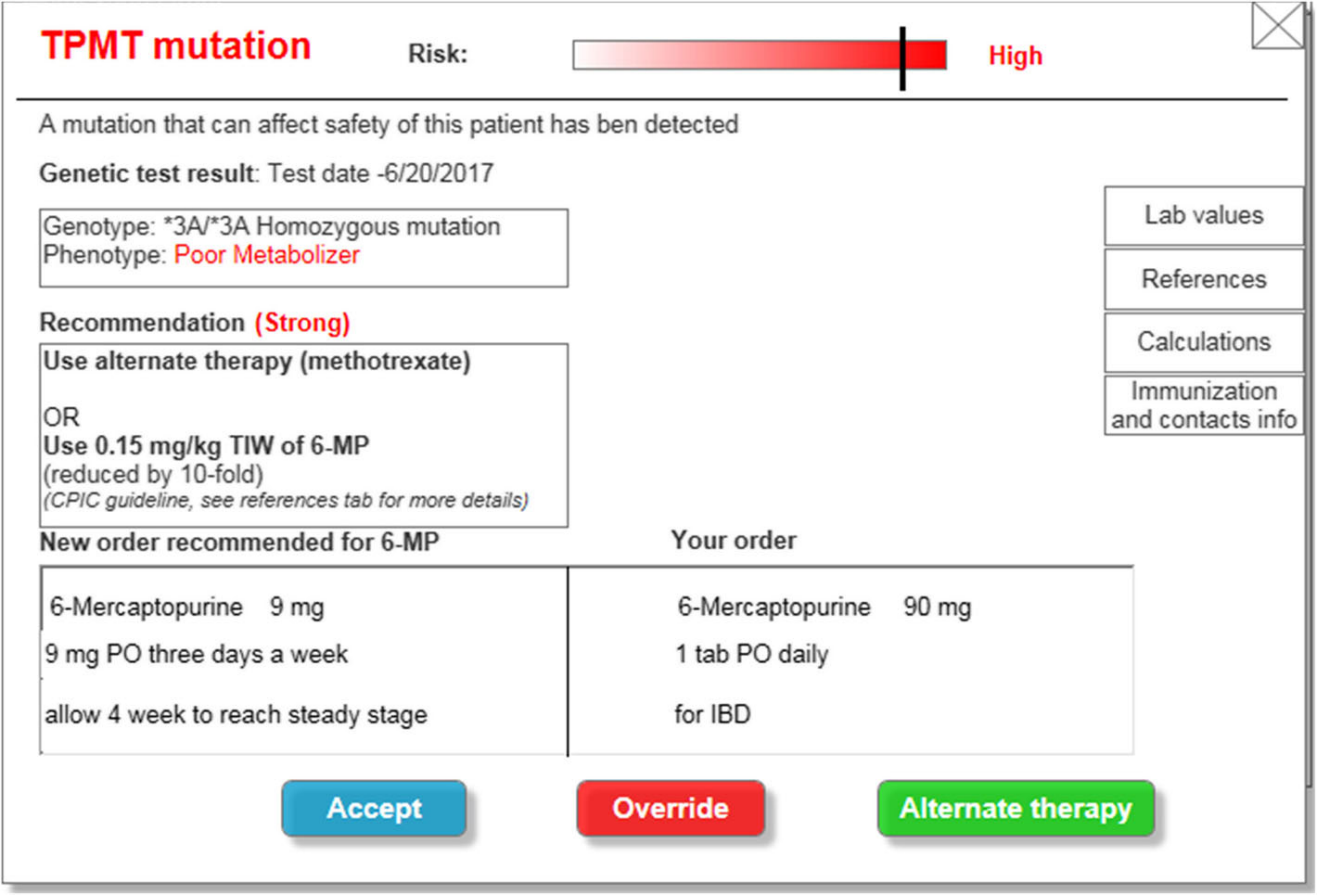
Screenshot of a PGx-CDS prototype interface. This is the 2nd version we used to conduct usability testing in phase II

Data from qualitative interviews and available literature [23–25] were used to inform the development of an electronic prototype of the PGx-CDS in a mock EHR (Additional file 1: S5.) The prototype was developed by a research pharmacist and a human-computer interaction expert. We created a list of key features to design a PGx-CDS for TPMT using both collected interview data and human factors principles (Table 1) These key features in Table 1 focused mainly on the display while interview data provided the content of the PGx-CDS prototype. In phase II of this study, we evaluated both the display and the content of the PGx-CDS prototype via usability testing. To facilitate iterative evaluation and redesign, the prototype was not implemented in a “live” EHR. This approach, which has been used in other studies [34, 35], allowed the PGx-CDS tool to be evaluated safely, separate from patient care, providing insight on how it can be improved prior to implementation. Instead, Axure RP 7 (Axure Software Solutions, Inc., San Diego, CA) was used to design the mock-up EHR as well as the prototype (Fig. 1) This was a cost-effective, rapid approach to help physicians visualize the potential design of a future PGx-CDS interface. Participants could interact with the prototype as if they were working with an actual PGx-CDS from the EHR. This prototype provided a realistic impression of what the PGx-CDS could look like in clinical practice. This also helped us identify early errors in the design. Finally, in clinical practice, a TPMT related PGx-CDS would need to support many types of prescribers for patients being treated for leukemia, IBD, or rheumatoid arthritis. Therefore, we chose to apply a universal design approach [36] for PGx-CDS with the goal of creating a CDS that can effectively support a wide range of primary care and specialty prescribers. A universal design approach is generally robust and often requires less training and education of end users [37].

**Table 1:** Key features of PGx-CDS display design

Subsequent design decision	Human factors principle(s) applied or *Interview results*
1. Important information is presented on a single screen	Hazard control hierarchy [26, 27]
2. Action buttons are color coded Blue for “Accept” button, red for “Override” button	Visual Cue [28]
3. Risk bar visualization indicates the danger of mutation Red for homozygous mutation, orange for heterozygous mutation The risk is categorized into high, moderate, low	Attention switch [29, 30]
4. Information is grouped into categories	Decision-making alignment Chunking [31] *Three main criteria are: genotype result, dosing adjustment recommendation from references, and new medication order recommended*
5. Recommendation(s) from the PGx-CDS is positioned so physicians can visually compare it to their current medication order	Proximity compatibility principle [32] Minimize user memory load [33]
6. Additional information (e.g., lab values, references, calculations, immunization such as flu shot, TB) and contact information) are presented as supplemental information (via clickable buttons) to avoid information overload	Minimize user memory load [33] Flexibility for user control of data display [33] *Interview results: PGx-CDS Content* Examples from the interviews: “*Less is more,*” *“One of the biggest things to know is the ANC [absolute neutrophil count], the platelet… so you can [evaluate the new order recommended]”* *“[Information from referencces] was condensed, it could be more helpful if you could turn things [certain lab values, calculations] off.”*
7. Supplemental information (e.g. calculation, lab values) can be view simultaneously with the main information	Minimize user memory load [33]
8. Physicians can choose between two options for reviewing reference sources: 1) access information online or 2) send references to their own, individual email	Flexibility [33] *Interview results: PGx-CDS content* Example: “*It would be nice to have [two options for reviewing reference sources] because most clinicians are not going to have time to read it right there*”

### Scenario development

Four standardized, fictitious patient scenarios were developed by a team consisting of a research pharmacist, a gastroenterologist, and an oncologist to test the usability of the PGx-CDS. Two scenarios were developed for oncology participants to represent leukemia patients, one with a homozygous and one with a heterozygous mutation, and two scenarios were developed for gastroenterologists (Additional file 1: S3) to represent patients with IBD. Patient scenarios were further refined based on feedback from a gastroenterology specialist and pilot testing with a primary care provider.

### Participant recruitment

Physicians from each healthcare system were recruited for a 30-min usability session. Eligibility criteria were the same as phase I: physicians were eligible to participate if they ever prescribed azathioprine or mercaptopurine in their clinical practice. Participants were consented in written prior to their sessions.

### Simulation procedure

One of two moderators, each with experience moderating usability sessions, conducted each session and read a scripted introduction (see Additional file 1: S1, moderator script) to maintain consistency across participants [35]. Participants were informed that the goal was to improve the design of the PGx-CDS. Participants were asked to complete two patient scenarios that were relevant to their practice and complete tasks as though they were prescribing for real patients (Additional file 1: S2.) A physical barrier separated the moderator from participants to help reduce the potential for bias [19]. Also, the moderator refrained from offering guidance to the participant during the usability session.

All participants completed tasks in the same sequence: one patient scenario with a homozygous mutation followed by one patient scenario with a heterozygous mutation. There was no time limit for each scenario. Participants were asked to ‘think aloud’ [38] by verbalizing their thoughts, reactions, and points of confusion as they interacted with the PGx-CDS while completing the scenarios. Afterward, participants were asked to respond to an online 19-item Computer System Usability Questionnaire (CSUQ) [39] via REDCap software [40] to gather their level of satisfaction with the PGx-CDS tool. Finally, the moderator concluded the session by conducting a debrief interview with the participant to collect additional feedback about the interaction.

We used an iterative method to test our prototype because our goals were to develop and rapidly refine a provider-centered PGx-CDS to help providers with their thiopurine prescribing decisions. Specifically, any usability errors identified during testing were used to enhance the design of the PGx-CDS prototype. A similar, iterative prototyping method has been successfully applied in other research studies of health information technology [41, 42].

### Data collection and outcomes measures

Qualitative usability data were collected via the “think aloud” technique with participants’ verbalizations captured by [38] Morae 3.3 (TechSmith Corp.) screen video and audio recordings. Additionally, qualitative data included information from debriefing interviews. Quantitative usability data were collected on four established measures of usability [41]: learnability, usability errors, efficiency, and satisfaction. Learnability and usability errors were evaluated by reviewing Morae® video recordings. Efficiency was measured from the time-stamped video recordings and included the amount of time the physicians spent completing each scenario as well as time spent on the PGx-CDS prototype interface. Finally, after working on the usability scenario, participants were asked to complete the CSUQ 19-item questionnaire [39] to evaluate their satisfaction with the PGx-CDS. Each participant completed the questionnaire once. In addition to capturing their overall perceptions of satisfaction of PGx-CDS, this questionnaire assessed their satisfaction for three main domains: system usefulness, information quality, and interface quality.

## Results

### Participants’ characteristics

Across both phases, 14 physicians (12 male; five from the VA and nine from the other MAHS) participated. Women accounted for 14% of the participants. The average age of participants was 39 years (range, 30 to 48 years). Table 2 summarizes the demographic information of participants.

**Table 2:** Demographic information. Physicians represented two specialties: oncology and gastroenterology

	Physicians				EHR experience	
	Specialty	Number	Adult	Pediatric	Years of VA EHR use mean (range)	Years using of other EHRs mean (range)
Phase I: Interviews	GI	5	3	2	4 (0–15)	6 (1–9)
	Oncology	5	2	3	6 (0–13)	6 (0–12)
Phase II: Usability testing	GI	2	1	1	8 (1–15)	5 (4–6)
	Oncology	5	4	1	6 (0–13)	3 (0–6)
Total	–	17^a^	10	7	5 (0–15)	5 (0–12)

*Abbreviations*: *GI* Gastroenterology, *VA* Veterans affairs, *CPRS* Computerized patient record system, *EHR* Electronic health record

^a^Represents 14 different providers since three providers participated in both interviews and usability testing

### Emergent themes from interviews

Interviews yielded 11 themes: *the need for PGx-CDS for TPMT; impact of PGx-CDS on clinical workflow; lab testing preferences; perceived barriers to PGx implementation; PGx-CDS content; PGx-CDS display; references within PGx-CDS; genetic result content; display of patients’ genetic results; PGx care coordination; and examples of related software and CDS systems*. Table 3 lists all 11 themes, overarching comments from participants, and example quotes. A table with definitions of each theme and comments from each specialty group is shown in the Additional file 1: S6.

**Table 3:** Themes identified from interviews

Theme	Comments from participants	Example quotes
A. Healthcare providers’ views on pharmacogenomics clinical decision support		
1. The need for PGx-CDS for TPMT	Physicians expected PGx-CDS to: • Notify them about TPMT genetic mutations • Provide clinical guidance • Help patients avoid serious side effects	*“I feel that every patient who is going to need mercaptopurine should have the genetic testing done and [PGx-CDS] is going to make it easier to interpret the enzymatic activity.” –* pediatric oncologist B *“We know what the goal ANC [absolute neutrophil count] should be, but how much [of the regimen] we need to adjust, that is something the CDS can help [with].” –* pediatric oncologist A *“[PGx-CDS] can help to make sure that we don’t start on a dose that’s too high that is going to cause significant myelosuppression.”-* pediatric oncologist A *“There is a lot of fatigue [that] plays into mercaptopurine dosing, but to have something [such as PGx-CDS] that would function to coordinate and assimilate information into one place, it would decrease fatigue over time.” –* adult oncologist B
2. Impact of PGx-CDS on clinical workflow	• PGx-CDS can be integrated into the workflow seamlessly since there is enough time to test and use genetic information prior to prescribing. • Physicians expressed that they would value real-time, integrated PGx-CDS to help them with prescribing and genetic testing decisions.	*“We don’t start mercaptopurine right away for the patient. Usually, it has a delay [phase 2 of treatment], so we usually have genetic test results before initiation mercaptopurine.”* – pediatric oncologist C *“If [PGx-CDS] does not pop up until the very first time you are prescribing the medication, it might be too late because you’re ready to prescribe the medication.” –* pediatric oncologist D *“I think the best way is when you are ordering the drug…[and] it is something that [is] right there in real-time [and] computer-based.” -* adult oncologist A
3. Lab testing preferences (genetic vs. enzymatic testing)	• Physicians stated that they order genetic labs based on the standard of care; there is no policy or specific requirement. • Physicians generally choose genetic or enzymatic testing based on the availability of tests at their hospital system and cost to the hospital and patients. • Providers (*n* = 6) generally preferred genetic over enzymatic tests	*“If I am going to reduce dosage, I don’t care that much about the phenotype [from the enzymatic test]. But if I have an abnormal genotype, [then] I absolutely want to know the enzyme activity. If it’s heterozygous, then I definitely want to know what the phenotype is.”* – pediatric GI A *“There is no requirement from the VA, It [test order] is more just based on clinician preference.” –* adult GI A *“Nowadays, [I] order genetic test[s] more often than enzymatic test[s].*” – pediatric oncologist C *“Enzymatic test[s] can help figure [out] the phenotype results that might not be related to genotypes…”* – pediatric oncologist C
4. Perceived barriers to PGx implementation	• Alert fatigue was the main concern with PGx-CDS • A PGx-CDS that is in a separate software package from the EHR was described as a barrier to use.	*“Alert fatigue is very real. [The EHR] is notorious in all of the alerts that pop up all the time and the inability for them to prioritize [alerts] is exhausting.”* adult oncologist A *“The biggest issue would be if you were unable to get it [PGx-CDS] incorporated into the EHR and it was something external that you have to pull up.”* – pediatric oncologist B
B. Important design features for PGx-CDS		
5. PGx-CDS content	• Physiciansrequested that PGx-CDS include information about the: ◦ Patients’ genotype ◦ Patients’ phenotype ◦ Meaning of the correlation between genotype and phenotype ◦ Potential medication risks ◦ Dosing recommendation ◦ Supporting references • Physicians expressed a desire to have all key information shown or readily accessible from the PGx-CDS screen to make their prescribing decision(s).	*“I want to know the genotype and phenotype because the heterozygote case is the one that is more complicated.”* – adult GI A *“It would be nice not just to present the enzymatic activity but to have an interpretation of the metabolites to give the clinician an idea of this specific mutation, [and] what are the risks for patients.” –* pediactric GI B *“If all stuff could get pulled out of their medical chart into this decision tool, you could retrospectively review all information, and it actually could be informative.” –* pediatric oncologist B
6. PGx-CDS display	Physicians expressed a preference for: • Concise presentation of information • PGx-CDS that is intrusive (not passive). E.g., as a pop up displayed in the middle of the screen • Lab values where trends across time can be easily viewed.	*“Pediatric oncology is very different than the primary care provider; we have more time…. we prefer to have the information right there in front. We see patients for 2–3 years for mercaptopurine, we see them often.” –* pediatric oncologist C *“Something [such as graphs] where you could actually see the trend and even a comment about adherence.” –* pediatric oncologist D *“I will say on the pop-ups, they come right in the middle [of the screen], and that is probably the best… I will pay more attention to it if it’s in the middle.” –* adult oncologist A
7. References within PGx-CDS	• Common resources that physicians currently use to find pharmacogenomic prescribing recommendations: ◦ Medical articles ◦ Protocols (e.g., from a clinical trial) ◦ UpToDate	*“Even with the homozygous result, we follow the protocol of [the] clinical trial upfront.” –* pediatric oncologist C *“I guess that [the idea of having reference information sent to your work email] would be helpful. In theory, you would only need to ever look at that once, but from an information standpoint, it would be handy to have it. That way people don’t have to search around.” –* pediatric oncologist B
8. Genetic result content	Physicians generally requested that PGx-CDS: • Provide a standard interpretation of genetic test results • Inform providers when a patient’s genetic test results are available in the EHR. • Provide reference ranges for lab results for comparison	*“[An] alert [in the EHR] that tells the provider if the [genetic] test has been done or not [before prescribing] would be beneficial.” –* pediatric oncologist E *“It is probably better to have it [genetic result] flagged in some way [to inform clinicians].” –* Adult GI A *“It would be nice to have a little line [that] said, the control test was normal, so this is a valid result.” –* pediatric GI A
9. Display of patients’ genetic results	Physicians offered several ideas about where to present patients’ genetic results in the EHR, including: • in the heading of the patient profile • on a new, pharmacogenomics tab • in the EHR’s patient history as part of the prescription details for pharmacists to double check when dispensing medications.	*“If someone was tested for [a] mutation and they were positive, if you could link [it] as an allergy, that would be good…”–* adult oncologist A *“I don’t know it could go under ‘medications’ or ‘past medication history’ [in the EHR]. There is no good heading for it, but maybe [under] ‘allergies’ [section]. Those allergies are typically linked to [medications] when I am prescribing.” –* pediatric oncologist 03 *“If there is a pharmacogenomic tab that has not only TPMT but other [genetic results], that would be even better.” –* adult GI B
C. Other		
10. PGx care coordination	Physicians suggested that PGx-CDS provide: • Options to follow up and coordinate genetic testing and results between healthcare providers • A tool to help communicate with providers outside of their own healthcare system • A tracking method within the EHR for outpatient care	*“For all patients who have the TPMT testing done, there can be a form that can be sent directly to our pharmacists so [s/he] can keep track of all patients for better communication.”* – pediatric oncologist D *“Lot[s] of times if we get referrals from elsewhere. They will often have the genotype performed, [and] we will use that information if we have it, but more often than not, we order it.” –* pediatric GI A *“We get a lot of patients from the outside, so finding a way to capture the information from the outside would be helpful… If there were some way that the TPMT status can be documented in our system from whatever came from the outside, that would be good.”* -adult GI A
11. Examples of related software and CDS systems	Physicians requested that PGx-CDS have similar functions and a format analogous to other CDSs currently implemented in their EHR	*“I have a software called VCM Chemo Manager that [is] built into the treatment plans. It does not force you to order them [lab tests], but it pops up [with alerts] when you go through the treatment plan.” –* adult oncologist A

*Ped GI* Pediatric Gastroenterology specialist, *Adult GI* Adult Gastroenterology specialist, *EHR* Electronic health record, *PGx-CDS* Pharmacogenomic clinical decision support

Some themes have been discussed elsewhere in the literature for other types of CDS [23–25, 43], therefore, the Results section below focuses on themes new to the literature that can benefit the implementation of PGx-CDS. These themes are presented into two overarching categories: 1) physicians’ views about PGx-CDS, and 2) key design features needed for PGx-CDS.

### Physicians’ views on pharmacogenomic clinical decision support

During interviews, all ten physicians expressed a high degree of interest in a PGx-CDS tool to help them with prescribing decisions for TPMT. They expected that PGx-CDS would help detect patients’ history of TPMT genetic mutation(s) when they prescribe either mercaptopurine or azathioprine and then provide guidance on how to “*adjust the dose*”. However, they emphasized that PGx-CDS needs to be incorporated into the current EHR rather than as a standalone software system and must operate in real-time to be effective.

Additionally, both specialty groups described the potential value of PGx-CDS in reducing their reliance on pharmacy-related resources. Oncologists indicated that the implementation of PGx-CDS might reduce the burden and workload for their pharmacy service. Specifically, physicians could use the PGx-CDS to look for new dosing recommendations and references rather than depend heavily on consulting the pharmacy service. In addition, one physician described that PGx-CDS can help assist with his/her work because “*there is a lot of [mental] fatigue [with] mercaptopurine dosing [because of irregular dosing regimens], but to have something [like a PGx-CDS] that would function to coordinate and assimilate information into one place, [it] would actually decrease [my] fatigue over time” (Pediatric oncologist C)*. Similarly, GI specialists who rarely prescribed TMPT medications or encountered this genetic mutation in patients shared that if PGx-CDSs were readily available in the EHR, they would “*be more inclined to use [the CDS]”* rather than consulting outside clinical references or primary medical literature.

### Important design features needed for clinical decision support

#### Content

Based on interview data, information that needs to be included in the PGx-CDS interface for TPMT decision-making are below, along with numbers of participants that expressed a need

- genotype (homozygous mutation or heterozygous mutation) (*n* = 5)
- phenotype (poor metabolizer, regular metabolizer, or high metabolizer) (*n* = 4)
- the meaning of the correlation between genotype and phenotype (i.e., whether the patient is at high/low/medium risk for side effects) (*n* = 6)
- potential medication risks for patients (i.e., myelosuppression, neutropenia); recommendation for dosing adjustment; (*n* = 9)
- Reference citations to support the dosing recommendation. Importantly, the prescriber needs to be able to trust the PGx-CDS and “*validate the recommendation with their own internal thought processes*.” (*n* = 8)

There are several additional features described as desirable by physicians, with evidence that some PGx-CDS design needs are unique to the type of physician specialist. Pediatric oncologists (*n* = 3) wanted to have clarification regarding the dosing calculation - how the new dosing recommendation was calculated since it can be based on the patient’s weight or body surface area - for medication dose adjustment. One pediatric oncologist also wanted PGx-CDS to provide guidance about the specific dosing scheme that is appropriate for the individual patient, since dosing regimens for azathioprine and mercaptopurine are not the same for each day of the patients’ medication regimen. For example, in 1 week, a patient can be taking one tab of azathioprine 50 mg for 4 days (M, W, F, Sun) and 1.5 tabs of azathioprine 50 mg for 3 days (T, Th, Sat) for a total of 375 mg/week. This complicated dosing regimen can become more confusing when additional, PGx-related dosing adjustment is needed. To account for the patient’s genetic profile, the PGx-CDS needs to provide clear guidance about this regimen. Also, a physician wanted PGx-CDS to help create a long-term dosing agenda for patients with complex regimens. This physician indicated that this type of support might save time for both the prescriber and pharmacist when counseling patients on the new dosing regimen.

Additionally, while pediatric oncologists (*n* = 2) expressed a preference to have a dose adjustment based on patients’ body surface area, gastroenterologists recommended that the PGx-CDS provide dose adjustment based on patients’ weight. Also, several physicians from both groups (*n* = 3) shared that since thiopurine medications are used for maintaining therapy when treating patients with either leukemia or IBD, the PGx-CDS should also recommend specific lab tests (e.g., white blood cells, hemoglobin) that should be ordered for monitoring medications. Physicians confirmed that lab values related to TPMT medications also are important information to present in the PGx-CDS. Additionally, physicians expressed a desire for the PGx-CDS to present the following options for medication management: neutrophil count as a lab value; ability to turn on/off certain options within the PGx-CDS content; and ability for the PGx-CDS to automatically generate recommendations for a new dosing regimen within the medication ordering screen of the EHR. Finally, if a patient presents with a TPMT homozygous mutation, gastroenterologist providers (*n* = 3) stated that they would be more likely to prescribe a different biologic agent rather than adjust the medication dose, therefore information like Tuberculosis test and vaccine information should be available to the prescriber through PGx-CDS so they can choose the alternate biologic medication more effectively.

#### Display

Physicians (*n* = 3) from both specialties expressed the importance of having clear and concise information on the PGx-CDS: “*less is more.*” Also, since the TPMT mutation can cause serious side effects for patients, they envisioned an interruptive, PGx-CDS pop-up in the middle of the screen to help them pay more attention.

When participants were asked whether they want to have an option to send the supporting reference(s) for PGx-CDS recommendations to their email account, and most (*n* = 9) were interested, including all five gastroenterologists. In contrast, oncologists generally wanted more opportunity to review patient information in real-time since “*pediatric oncology is different than the primary care setting, we have more time”*. Therefore, they preferred to have all information, including reference content, presented on the PGx-CDS interface to help them make prescribing decisions.

### Usability testing

#### Efficiency

**Fig. 2:**
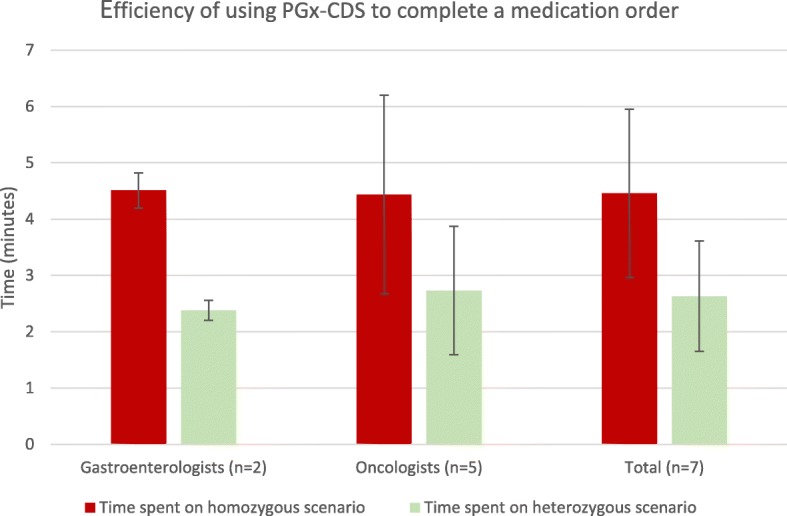
Efficiency of using PGx-CDS to complete a medication order

Efficiency was measured by the time each physician spent to complete the medication orders (see Fig. 2) All participants (*n* = 7) successfully completed all assigned patient scenarios for the usability tasks without help from the moderator.

#### Satisfaction

Satisfaction ratings were high across participants (Table 4). One participant (#5), however, rated the ‘interface quality’ relatively low (mean 3.3).

**Table 4:** Satisfaction scores for the PGx-CDS interface^a^. Data for individual participants are presented as means

	System usefulness (items 1–8)	Information quality (items 9–15)	Interface quality (items 16–18)	Overall satisfaction (item 19)
Participant 1:	6.0	4.8	4.7	5.0
Participant 2:	7.0	7.0	7.0	7.0
Participant 3:	7.0	7.0	7.0	7.0
Participant 4:	6.1	6.5	6.0	6.0
Participant 5:	7.0	7.0	3.3	5.0
Participant 6:	6.0	6.0	6.0	6.0
Participant 7:	7.0	7.0	7.0	7.0
Gastroenterologists: (*n* = 2) median (range)	7.0 (6.0–7.0)	7.0 (4.8–7.0)	6.0 (4.7–7.0)	6.0 (5.0–7.0)
Oncologists: (*n* = 5) median (range)	6.5 (6.0–7.0)	6.5 (60–7.0)	6.5 (6.0–7.0)	6.5 (6.0–7.0)

^a^Participants rated each item on a 7-point Likert-type scale where 1 = strongly disagree and 7 = strongly agree. Results are shown as mean for each individual and median (range) for each physician group

During the usability sessions, participants across specialties expressed high satisfaction with this prototype. They were especially satisfied with the visual design of the alert (*n* = 5). One adult GI specialist mentioned*, “I like that the [warning] is red to alert me about the TPMT mutation. It catches my attention.” Similarly,* an adult oncologist stated, “[the] a*lert is visually well designed, it gets your attention, things that [are] important are in red, it gives you some options [to make decision].”*

#### Usability errors

We highlight four usability errors that were uncovered during usability testing. First, several participants (*n* = 3) mentioned they were unclear whether the “*Accept*” button approved the PGx-CDS recommendation or approved their original medication orders. A pediatric oncologist stated, “*I assume ‘Accept’ will accept a new order but it is not clear. ‘Accept new order’ will be more clear – pediatric oncologist E.*” Another participant suggested changing the label to “*Accept recommendation*. – *Adult oncologist C*” Also, the initial button labeled “*Others*” also was unclear for several participants (*n* = 3). An adult GI specialist mentioned, “*I like the ‘Other’ button but it does not necessarily tell me what that is.- Adult GI C*” Thus, we replaced it with “*Immunization and contact info*” to aid clarity during usability testing. Second, the earlier version of the PGx-CDS prototype did not have a cancel button to allow participants to get out of CDS without further action. This hinders the control and freedom of prescribers when ordering medications. We added this option to the prototype during the usability testing period after two participants completed usability testing. Third, when participants used the “Override” button from the CDS interface, they stated that it lacks a warning message to prompt them about their potential dangerous actions of overriding the recommendation. An adult GI specialist commented, *“[PGx-CDS] should have a second level of warning such as, ‘This patient is at a very high risk of severe leukopenia, are you sure you want to expose patient to that risk?’- Adult GI D*.” Another adult GI specialist stated, “*For [the] override, you should build something like, ‘Are you sure you want to override this recommendation?’. It can be malpractice if overriden.- Adult GI D*” Finally, two providers mentioned that the CDS interface did not provide a patient’s weight or body surface area (BSA) to help providers confirm the dosing calculation. A pediatric oncologist mentioned*, “This CDS is based on weight, we used BSA to calculate the dose. – Pediatric oncologist F”* This suggestion was noted for future improvement.

## Discussion

This is one of the first studies to employ a user-centered design approach to develop a clinical decision support tool for pharmacogenomics, specifically for TPMT medications. Key contributions of this research include the following: 1) application of human factors engineering to design a PGx-CDS for TPMT, 2) analysis of prescribers’ information needs and developed a PGx-CDS prototype using information collected from practicing providers, and 3) evaluation of a prototype PGx-CDS via usability testing, which could inform future implementation of PGx-CDS in EHRs to aid in the care of patients. While a user-centered design approach has been used to develop many other types of CDS based on the literature [44, 45], it appears that user-centered design approaches have been underutilized for few PGx-CDSs, including TPMT biomarker for azathioprine, or mercaptopurine. As discussed in the introduction, pharmacogenomic clinical services are still at an early stage of development. It is important to collect prescribers’ information needs and design a PGx-CDS system that supports prescribers’ workflow for pharmacogenomic prescribing. Therefore, the development of new PGx-CDSs using a combination of human factors engineering and information collected from end-users is critical for the successful adoption and implementation of pharmacogenomic services.

This study aligns with some information found in prior literature. For instance, our results agree with two studies which found that PGx-CDS should present dosing guidelines and recommendations to users rather than just information about the potential harm of the adverse interaction [15, 23]. Since the numbers of biomarkers and availability of genetic tests are increasing, it is unsustainable to expect providers to immediately recall guideline recommendations and how to interpret all pharmacogenomic results. Our results also agree with Devine et al. that PGx-CDS should include a summary of the literature related to PGx mutations, dosing recommendations, and the ability for end-users to verify the recommendation sources [15].

Despite these commonalities, our study was unique compared to prior research in that it applied a human factors approach to develop the PGx-CDS prototype for TPMT. Specifically, the development of our PGx-CDS was informed by “the four pillars of successful user-interface development,” published by Shneiderman et al. [46] to increase the chance of successful implementation. These four pillars are 1) user interface requirements, 2) guidelines documents and processes, 3) user-interface software tools, and 4) expert reviews and usability testing. The ‘user-interface software tools’ pillar was described in our Methods section. Below, we use the remaining three pillars to organize the discussion of our study results.

### User interface requirements

As described by Jacko [47], clearly specifying user requirements is key to successful interface development. In this study, the process of a hybrid analysis of physician interviews allowed us to identify specific aspects that they perceived as important design features for PGx-CDS. We organized these design features into PGx-CDS “content” and “display.” The information we gather about PGx-CDS content and display allowed us to include essential information into PGx-CDS interface prototype that providers needed to make prescribing decisions. Additionally, supporting references for the PGx-CDS recommendations can help gain providers’ trust when they make prescribing decisions. These features were then separated further for distinct comments between two subspecialties. This allowed us to add options to the PGx-CDS prototype design to help support the needs of both physician specialties. For example, the PGx-CDS interface (Fig. 1) gave physicians options to directly access the full reference details for recommendations (preferred by oncologists) or to send those references to the physicians’ work email addresses (preferred by gastroenterologists). The PGx-CDS interface we designed received high satisfaction scores from both types of physician specialists in ‘information quality’ and ‘interface quality’ (Table 4), which is likely because we gathered and applied design requirements directly from users via interviews.

### Guidelines documents and processes

We used our findings to create a summary of PGx-CDS design recommendations (Table 1) to assist with new PGx-CDS development for TPMT. This table was generated from both interview data and human factors principles to align the PGx-CDS with physicians’ workflow and needs. These design recommendations are not only applicable for TPMT but may also inform the development of PGx-CDS for other pharmacogenomic biomarkers. In addition, they are not EHR dependent. CDS developers can follow these recommendations to develop PGx-CDS interfaces that align with the requirements and constraints of each specific EHR system.

### Expert review and usability testing

We conducted formal usability testing with physicians to evaluate the design of our novel PGx-CDS prototype interface. Overall, providers were able to learn how to use the new PGx-CDS effectively and were highly satisfied with the interface and design of the PGx-CDS for TPMT. Satisfaction rates (Table 4) were high in all three aspects of the PGx-CDS prototype (system usefulness, information quality, and interface quality). These findings demonstrate the enthusiasm of physicians for this type of PGx-CDS and the value of applying a user-centered approach in the development of a new PGx-CDS prototype. Providers will be more likely to adopt a new CDS when they are satisfied with the design of the system [48].

This study has several limitations. First, it is possible that our sample is affected by selection bias since we did not randomly select participants. Providers who were more interested in PGx-CDS may have been more willing to participate in the interview and usability evaluation than those not interested in PGx-CDS. This bias may have contributed to our positive findings for satisfaction with the PGx-CDS. We took steps to reduce other potential biases by standardizing interview and usability procedures. Second, providers were informed that patient scenarios were fictitious, and they might not respond to PGx-CDS recommendations in the same manner as they would for real patients. Third, we applied the think-aloud technique, which can sometimes lengthen time measurements. Therefore, the time physicians spent on the PGx-CDS tasks may be greater in our study than in actual clinical practice. Fourth, only 14% of our participants were women in this study. While we attempted to recruit as many women participants as possible from all eligible participants, the small number of female providers is a limitation of our study. Finally, we only recruited seven participants for usability evaluation. The small sample size limited the number of usability findings. Nevertheless, work by Nielsen et al. indicates that 5 participants can identify up to 80% of major usability errors [49].

### Future directions

The results of this study can provide further guidance in the development and enhancement of other PGx-CDS interfaces, such as PGx-CDS for clopidogrel, or CYP2D6. The key features we identified in this project (Table 1) can serve as a reference for new studies to evaluate PGx-CDS interface design as well as the development of new PGx-CDSs. We plan to continue this research line with PGx-CDSs development in an actual functional system at a well-established institute in the US. Specifically, we will evaluate and compare the current implemented PGx-CDS interfaces at that institute with newly developed PGx-CDS tools that utilized the guidance from this project. Those result can help identify the strengths and weaknesses of different PGx-CDS designs in clinical practice. Also, our findings can inform related approaches to develop other PGx-CDS workflows in the EHR, such as passive PGx-CDS that appears to the side of the screen, for example, or a mix of passive and interruptive PGx-CDS. Finally, user-centered approaches can be used to develop new PGx-CDS that utilize not only pharmacogenomics results but also other factors such as the patient’s profile in the EHR and drug-drug interactions from their medication list to provide further guidance in pharmacogenomic medication decision-making for prescribers.

## Conclusion

This is one of the first studies to apply user-centered design to develop a PGx-CDS tool for physicians who prescribe TPMT medications. This research resulted in a satisfying PGx-CDS interface prototype for physicians, which may be useful to patients receiving TPMT medication treatment. Our results suggest that providers are interested and in need of PGx-CDS for TPMT to improve treatment safety and efficacy. This study provides further guidance to help with the development of clinical decision support for pharmacogenomics, especially for the TPMT biomarker.

## Supplementary information

**Additional file 1: S1.** Moderator script. **S2.** Task sheet for oncologist participants. **S3.** Example of a case for gastroenterologists. **S4.** Interview questions. **S5.** Screenshot of a mock-up electronic health record. **S6.** Definitions and comments from each group.

## Data Availability

The datasets used and/or analyzed during the current study are available from the corresponding author on reasonable request.

## Abbreviations

BSA: Body surface area
CDS: Clinical decision support
CPRS: Computerized Patient Record System
CSUQ: Computer System Usability Questionnaire
EHR: Electronic health record
GI: Gastroenterology
HSR&D: Health Services Research and Development
IBD: Inflammatory bowel disease
MAHS: Major academic healthcare system
PGx-CDS: Pharmacogenomic clinical decision support
TPMT: Thiopurine methyltransferase
VA: Veterans Affairs
