# Supplementary material for: Utilizing a user-centered approach to develop and assess pharmacogenomic clinical decision support for thiopurine methyltransferase
Source: BMC Med Inform Decis Mak. 2019 Oct 17;19:194. doi: 10.1186/s12911-019-0919-4 (PMC6798472; doi:10.1186/s12911-019-0919-4)
Supplement: Supplementary file 1 — Additional file 1: S1. Moderator script. S2. Task sheet for oncologist participants. S3. Example of a case for gastroenterologists. S4. Interview questions. S5. Screenshot of a mock-up electronic health record. S6. Definitions and comments from each group. [file 12911_2019_919_MOESM1_ESM.docx]

SUPPLEMENTAL DOCUMENT

1. **Moderator script**

This is a research study to assess a technology that helps providers when prescribing medications called “Pharmacogenomics clinical decision support tool” or PGX-CDS. Your participation in this 30 minutes section will help us improve the design of the tool

As you complete the **2 cases** provided in our session today, please try to work as though they are part of your normal work tasks even if some tasks are slightly outside of your normal scope of practice.

- You can order, change, renew, cancel, or discontinue ANY of the medications. Please adjust medications and respond to any alerts, as needed, as though you were prescribing for an ACTUAL patient.
- If you ever receive an alert and want to order a different medication, please just verbally state the name of the medication that you would order.
- If you need to order a new lab test, including genetic lab, please just verbally state the lab test you would like to order

Some medications may be different than what you normally prescribe, but please complete the scenarios as best as you can. Since this is a simulation study, you may feel as though you have less autonomy, and some things may feel different than a normal practice. To complete these tasks, you will use a mock-up electronic health record. Patient information provided is also fictitious information.

Please be honest in your feedbacks as your opinions are really important to help us improve the design. We are evaluating the tool, not you or your clinical knowledge. To help us assess the design, I will ask you to ‘think aloud’ for some patients. You do NOT need to ‘think aloud’ for the entire scenario, only as you encounter alerts and make decisions related to alerts. **When you ‘think aloud’, please verbalize your reactions to alerts, thought process, and things that you find frustrating, surprising, confusing, and also things you like about the alerts**. “Think aloud” may seem unnatural or uncomfortable since you might use to working silently. If you forget, I will gently remind you to verbalize what you are thinking. I am going to show you an example of the Think Aloud Procedure. Please lick on the file name “think aloud” and watch the video.

Now, I am going to sit in a separate section in the lab where I will view your screen remotely. I will let you know when you can open the folder and begin.

During the session, you may ask questions and I will purposefully not respond so that I can avoid biasing the session. You may now open the paper folder and begin the first task. Please remember to read task aloud and think aloud as you go.

1. **Task sheet for oncologist participants**

**CASE 1**

Goal: To prescribe a maintenance therapy for a patient diagnosed with ALL

Janis Brown is a 17-year-old female student who are being treated for **acute lymphocytic leukemia**. JB went through induction and consolidation phases and currently need to be on a maintenance chemotherapy program.

PMH: none

**Begin to order 6-mercaptopurine for this patient using Case 1 EHR provided on the desktop**

Usual initial normal dose of 6-mercaptopurine: 1.5mg/kg to 2.5mg/kg

For research purpose, assume 6-mercaptopurine is available at all dosing strengths

Please state “I am done with case 1” after finishing prescribe the medication and close the “Case 1 EHR” webpage

**CASE 2**

You encounter Jimmy Shen today to follow up with him about his ALL. Jimmy is a 14 years old children who recently went through remission treatment and need a new therapy for maintenance.

PMH: none

Weight **50 kg**

Patient comes back for a follow up visit. **You decided to prescribe 6-MP (mercaptopurine) for maintaining dose (normal initial dose 1.5mg/kg)**

For research purpose, assume 6-mercaptopurine is available at all dosing strengths

When you are finish with case 2, please close the case 2 EHR webpage and tell the moderator

1. **Example of a case for gastroenterologists**

Loren Willis is a 15-year-old student who reports that she has been in good health until the last few months, when she started to experience increasing abdominal pain and diarrhea. Loren is diagnosed with ulcerative colitis, an inflammatory bowel disease. She has been treated with prednisone and 5-ASA (5-aminosalicylic acid) suppositories and response well with steroid

PMH: none

FH: no family history of GI

SH: no history of alcohol, tobacco or drug use

Lab

| 4.9 | 13.1 | 279 |
| --- | --- | --- |
|  | 39.4 |  |

Weight: 60kg

| 141 | 106 | 9 | 85 |
| --- | --- | --- | --- |
| 4.4 | 28 | 0.6 |  |

ALP 53 ALT 24 AST 26 TBIL 0.23 CRP <0.29

**You begin to order 6-mercaptopurine for this patient.**

Usual initial normal dose of 6-mercaptopurine: 1.5mg/kg.

For research purpose, assume 6-mercaptopurine is available at all dosing strengths

1. **Interview questions**

Goals:

- Identify their perceptions/awareness about pharmacogenomic clinical decision support tools
- Understand barriers that can affect implementation/effectiveness of CDS
- What type of information and how such information should be presented in an alert (both active and passive)?

Introductions:

We are conducting research to understand clinician perceptions about pharmacogenomic clinical decision support tool. Specifically, we want to understand what information is needed to add to a clinical decision support tool for TPMT when prescribing azathioprine, mercaptopurine, or thioguanine. We are interviewing all clinicians who have prescribed either one of those three medications mentioned above to obtain more clues. Results from these interviews may be used to help us design our pharmacogenomic clinical decision support tool prototype. A usability testing for this prototype will be conducted later as part of the study. If you are interested to participate in this usability test, please let us know.

There are two parts of the interview for today. First, we will ask some questions about your background. Then, we will ask some questions about your thought with clinical decision support tool. If you do not want to answer a question, you can ask me to skip the question. At any time, you can also ask me to stop the interview for any reason without any penalty.

We will also record the discussion since it’s hard to write down everything during the whole session. The tapes will be transcribed and treated as confidential and shared only with the research team. Please let us know if you do not want to be recorded at any time during the interview and we will turn off the record.

*Demographic information:*

1. What is your current VA position?
2. What degrees or certification do you hold?
3. How long have you use CPRS to prescribe medication
4. What is your age?
5. Gender
6. How often do you prescribe AZA, MP, or TG?

Daily, weekly, monthly, a few times a year

1. How often do you order genetic testing for TPMT?
2. During prescribing process for AZA, MP, or TG, do you consult external resources to make clinical decision if genetic information for TPMT available?
   1. What resource do you use and why?

*Qualitative interview questions*Have you ever use any pharmacogenomics, computerized clinical decision support? Please describe

1. What concerns, if any, do you have when one of your patients have TPMT mutation?
2. What is your perception and interpretation of the meaning of TPMT pharmacogenomic clinical decision support tools?
3. If the computer system provided decision support for pharmacogenomics would you use it in your clinical practice? Why or why not?
   1. How might such a tool be helpful to you?
   2. What barriers do you foresee for incorporating the tool into your work?
   3. Would you like such a system work similar to an alert system?
   4. What information would you like to see presented in a PGx-CDS?
4. Would you want to have genetic guidelines, extra information (research studies, interpretation…) sent to your email as an option of CDSS?
5. If genetic testing results are incorporated into the EHR, where would you like to see them displayed?
   1. Genetic test results could potentially be displayed on the cover sheet, near the patient’s allergies. What are your thoughts on that?
6. Would you like to participate in usability test for this PGx-CDS later on? If yes, we will contact you later to schedule a test when it is available.
7. **Screenshot of a mock-up electronic health record**


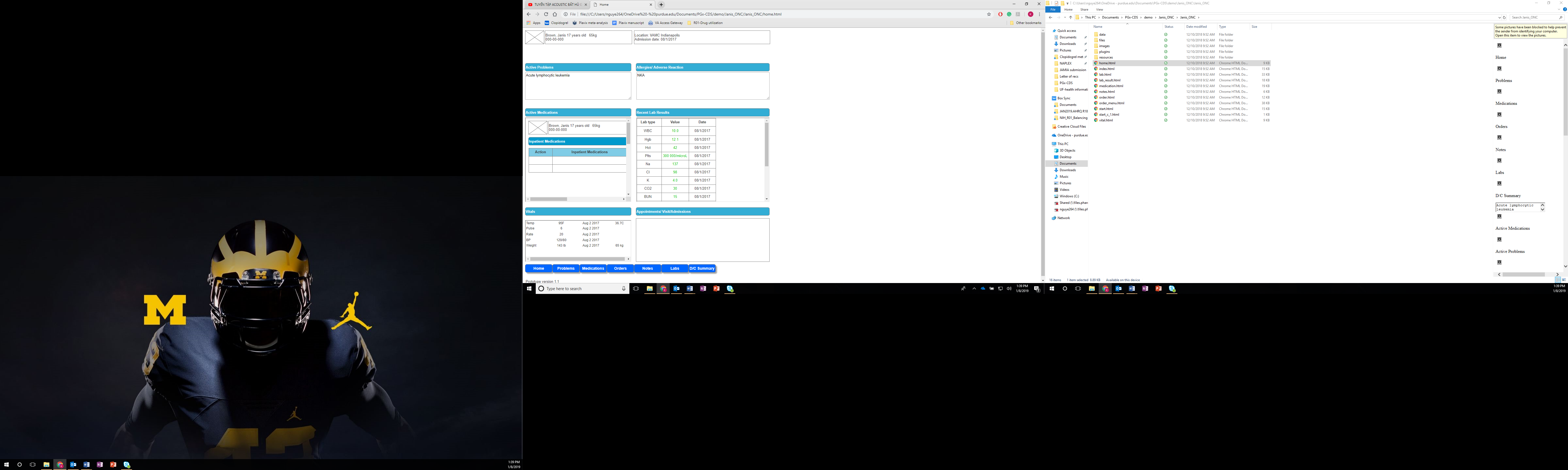


**6. Definitions and comments from each group**

| **Theme** | **Definition** | **Oncology comments** | **Gastroenterology comments** |
| --- | --- | --- | --- |
| 1. The need for PGx-CDS for TPMT | Motivational factors as described by physicians and reasons that they would want to use PGx-CDS in clinical practice | - Requested that the PGx-CDA provide a guideline to standardize the dose adjusting process - PGx-CDS may reduce the burden on pharmacy service | Anticipate that PGx-CDS for TPMT will:   - Improve evidence-based practice - Align prescribing decisions with treatment guidelines - Reduce their workload and improve prescribing accuracy, especially for infrequent users |
| 2. Impact of PGx-CDS on clinical workflow | Information related to the timing, navigation, or the logic of PGx-CDS within clinical practice | - PGx-CDS should provide dosing recommendations prior to initiating thiopurine treatment - PGx-CDS should remind prescribers to order genetic tests prior to the initial? prescribing decision | - Prefer to wait for genetic results before prescribing thiopurine medications, in order to prevent unwanted side effects |
| 3. Lab testing preferences (genetic vs. enzymatic testing) | Physicians’ preference, rationale, and factors for choosing a specific type of lab test: either a genetic test to assess the patient’s genotype versus an enzymatic test for the patient’s phenotype | N/A | - Expressed concern that enzymatic test might be affected by the patient’s concurrent medications - If the patient has a heterozygous genotype, a provider wants to do an extra enzymatic test to determine the TPMT activity level |
| 4.Perceived barriers to PGx implementation | Providers’ stated reasons regarding potential barriers, or factors that might negatively affect the implementation of PGx-CDS. | - Expressed that implementation might be a challenge for pediatric populations since, currently, most PGx recommendations and guidelines are only available for adult patients. | - Providers might not be receptive to a new PGx-CDS |
| 5.PGx-CDS content | Providers gave examples of instructions, recommendations, or wording that they wanted to see presented by the PGx-CDS | N/A | N/A |
| 6.PGx-CDS display | Providers’ comments related to visual presentation of PGx-CDS such as color, picture, shape, location, etc. | - Requested access via PGx-CDS to all supporting references so they can confirm the recommendation | - Preferred to have supporting references for the PGx-CDS sent to their email |
| 7.References within PGx-CDS | All information related to supporting references for PGx-CDS such as: the format of recommendation within the PGx-CDS, or factors used to make the decision whether to trust or follow the CDS recommendation | N/A | - More likely to select an alternative medication, rather than adjusting dosage if the patient’s genetic testing results show low TPMT activity |
| 8. Genetic result content | Instructions, recommendations, or wording from providers that should be presented in the genetic lab results | N/A | N/A |
| 9. Display of patients’ genetic results | Providers suggestions related to the visual presentation of data for genetic lab results. Visual suggestions could include items such as: color, picture, shape, location, etc. | N/A | N/A |
| 10. PGx care coordination | Providers commented on care coordination with other professionals to manage pharmacogenomics prescribing and patient care | N/A | N/A |
| 11. Examples of related software and CDS systems | Providers mentioned software or CDS systems as examples to inform future PGx-CDS. | N/A | N/A |
